# Cancer Stem Cell Marker DCLK1 Correlates with Tumorigenic Immune Infiltrates in the Colon and Gastric Adenocarcinoma Microenvironments

**DOI:** 10.3390/cancers12020274

**Published:** 2020-01-22

**Authors:** Xiangyan Wu, Dongfeng Qu, Nathaniel Weygant, Jun Peng, Courtney W. Houchen

**Affiliations:** 1 Department of Medicine, University of Oklahoma Health Sciences Center, Oklahoma City, OK 73104, USA; xiangyanwu@outlook.com (X.W.); dongfeng-qu@ouhsc.edu (D.Q.); 2 Academy of Integrative Medicine, Fujian University of Traditional Chinese Medicine, Fuzhou 350122, China; nweygant@gmail.com; 3 Department of Veterans Affairs Medical Center, Oklahoma City, OK 73104, USA; 4 Peggy and Charles Stephenson Cancer Center, Oklahoma City, OK 73104, USA; 5 Fujian Key Laboratory of Integrative Medicine in Geriatrics, Fujian University of Traditional Chinese Medicine, Fuzhou 350122, China

**Keywords:** tumor immunity, GI cancer, DCLK1, biomarker and adjuvant targets

## Abstract

Immunotherapy that has proven efficacy in several solid cancers plays a partial role in improving clinical outcomes of advanced gastrointestinal (GI) cancers. There is an unmet need to find new immune-related therapeutic targets. Doublecortin-like kinase 1 (DCLK1) marks tuft cells which are recognized as cancer-initiating cells and regulators of the type II immune response, and has been studied for its role in many cancers including colon and gastric cancers, but its role in tumor immunity remains unexplored. In the current study, we analyzed colon and gastric cancer RNA sequencing data from 283 and 415 patients, respectively, from The Cancer Genome Atlas (TCGA). High DCLK1 expression predicted the worse clinical outcomes in colon and gastric cancer patients and correlated with increased immune and stromal components. Further analysis indicated that DCLK1 was strongly linked to infiltration of multiple immune cell types, especially TAMs and Treg, and strongly correlated with increased CD8+ T cell inhibitors TGFB1 and CXCL12 and their receptors, suggesting it may contribute to TAM-mediated inhibition of CD8+ T cells. Interestingly, we found that DCLK1 was a prognostic biomarker in left-sided colon cancer, which has worse outcomes and demonstrates a reduced response to existing immunotherapies. In conclusion, our results demonstrate that DCLK1 is linked with functional regulation of the tumor microenvironment and may have potential as a prognostic biomarker and adjuvant target to promote immunotherapy sensitivity in colon and gastric cancer patients.

## 1. Introduction

Gastrointestinal (GI) cancers are exceptionally common with colorectal (CRC) and stomach cancer ranking third and fourth, respectively, worldwide [1,2]. CRCs arise from a polyp beginning with an aberrant intestinal crypt [3] and stomach cancers commonly arise in a background of intestinal metaplasia [4]. Similarities include shared somatic mutations (e.g., CTNNB1, KRAS) [5,6,7] and key roles for exposure to pathogens and inflammatory injury in tumor initiation [8,9,10]. In addition, colorectal and stomach epithelium contains LGR5+ stem cells and DCLK1+ tuft cells, both of which have been linked to human GI cancer initiation and progression, and identified as GI cancer cells-of-origin using mouse models [11,12,13,14]. Based on these shared characteristics, it may be possible to identify similar targeting strategies to improve colon and stomach cancer outcomes.

Several treatment strategies are employed in GI cancer including surgery, chemotherapy, radiotherapy, and molecularly targeted therapy. However, the survival of patients with advanced GI cancer remains poor [15,16]. Immune-related mechanisms play an important role in GI cancer, and immunotherapies are emerging as an effective treatment option against several types of cancer, including specific GI cancer subtypes such as those with high microsatellite instability (MSI-H). MSI-H GI cancers have relatively high numbers of mutations, leading to the production of neoantigens which can support extensive immune infiltration and sensitize tumors to immune checkpoint inhibitors [17]. Immunotherapies such as CTLA4, PD-1, and PD-L1 inhibitors show the most promising antitumor effects in malignant melanoma and non-small-cell lung carcinoma [18,19]. However, the current generation of immunotherapies (e.g., CTLA4 mAb) has shown poor clinical efficacy in metastatic colorectal and gastric cancers, while PD-1 and PD-L1 mAbs may result in partial responses in this context [20,21,22]. Therefore, there is an urgent need to identify novel immune-related therapeutic targets in GI cancers.

CD8+ cytotoxic T cells are key players in antitumor immunity. High CD8+ T cell infiltration is significantly correlated with better survival and there is mounting evidence to suggest that defective T cell migration into and within tumors constitutes a resistance mechanism to immunotherapy [23,24,25]. In the tumor microenvironment (TME), stromal cell-like tumor-associated fibroblasts and immune cells communicate with each other to decide the fate of tumor cells. For example, cytokines IL-4 and IL-10 secreted by tumor cells or T cells can polarize macrophages to their M2 state in which they are capable of secreting TGF-B1 and CXCL12 to inhibit CD8+ T cell function in support of tumor cell survival and metastasis [26,27,28]. These macrophages are termed tumor-associated macrophages (TAMs), which are key determinants of prognosis and the efficacy of immunotherapy [29,30]. In a TAM-enriched TME, CD8+ T cell-dependent immunotherapies (e.g., anti-PD-1) are likely to have little effect against progression [30,31]. Understanding the functional role of TME components in T cell exhaustion and reversing this state are keys to improving immunotherapy. However, the precise mechanisms involved in TAM-mediated inhibition of CD8+ T cells remain unclear.

Doublecortin-like kinase 1 (DCLK1) is a marker of GI tuft cells and has been reported to play an important role in cancer initiation and development in various cancers [14,32,33]. Previous studies show that DCLK1 is overexpressed in gastric, pancreatic, colon, renal, and other cancers [34,35], and plays a functional role in tumor initiation, growth, metastasis, epithelial-mesenchymal transition (EMT), and cancer stemness. A recent study from Westphalen et al. demonstrated that long-lived DCLK1+ tuft cells serve as colon-cancer-initiating cells when combing oncogenic mutation such as loss of APC and inflammatory insult [14]. Several studies also indicated that downregulating DCLK1 expression or inhibiting its kinase activity leads to growth arrest of colorectal, pancreatic, and renal cancers [36,37,38] as well as decreases metastasis of colorectal and pancreatic cancers [39,40]. Mechanistically, DCLK1 regulates NOTCH, NF-kB, KRAS, and WNT molecular signaling pathways that promote cancer growth and progression as well as support EMT and stemness of cancer cells [41,42,43,44]. In addition, DCLK1 regulates several miRNAs such as miR-let-7a and miR-200a which are known to be involved in tumor growth and EMT [36,38]. Most importantly, recent results of cell lineage-tracking analysis in mice suggested that DCLK1 is specific to intestinal CSCs and not expressed in normal stem cells, making targeting DCLK1 with novel therapeutic strategies a promising approach [45]. Recent findings unequivocally show that DCLK1+ tuft cells regulate the type II immune response in a variety of infection and inflammation models [46,47,48,49,50,51,52,53,54]. In the small intestine, taste receptors on DCLK1+ tuft cells detect threats and secrete IL-25, which binds its receptors (IL-17Rb) on type 2 innate lymphoid cells (ILC2s), inducing IL-4/13 secretion, which activates IL-4 receptors and induces tuft and goblet cell hyperplasia, which can initiate tumors and support progression and metastasis when harboring mutations [14,46,47,48,55], but few studies have assessed DCLK1′s role in tumor immunity. In this study, we assessed DCLK1 for its role in the TME and as a prognostic marker in colon and gastric cancer using RNA-Seq data from 283 and 415 patients, respectively. We found that DCLK1 expression correlated with increased stromal and immune components in the TME. Further analysis indicated that DCLK1 was strongly linked to infiltration of multiple immune cell types, especially TAMs and Treg, and strongly correlated with increased CD8+ T cell inhibitors TGFB1 and CXCL12 and their receptors, suggesting it may be a contributor to TAM-mediated inhibition of CD8+ T cells. Finally, analysis of patient outcomes demonstrates its potential as a prognostic biomarker and therapeutic target for gastric and left-sided colon cancers.

## 2. Results

### 2.1. High Expression of DCLK1 is an Independent Prognostic Factor in Colon and Gastric Adenocarcinoma

**Figure 1:**
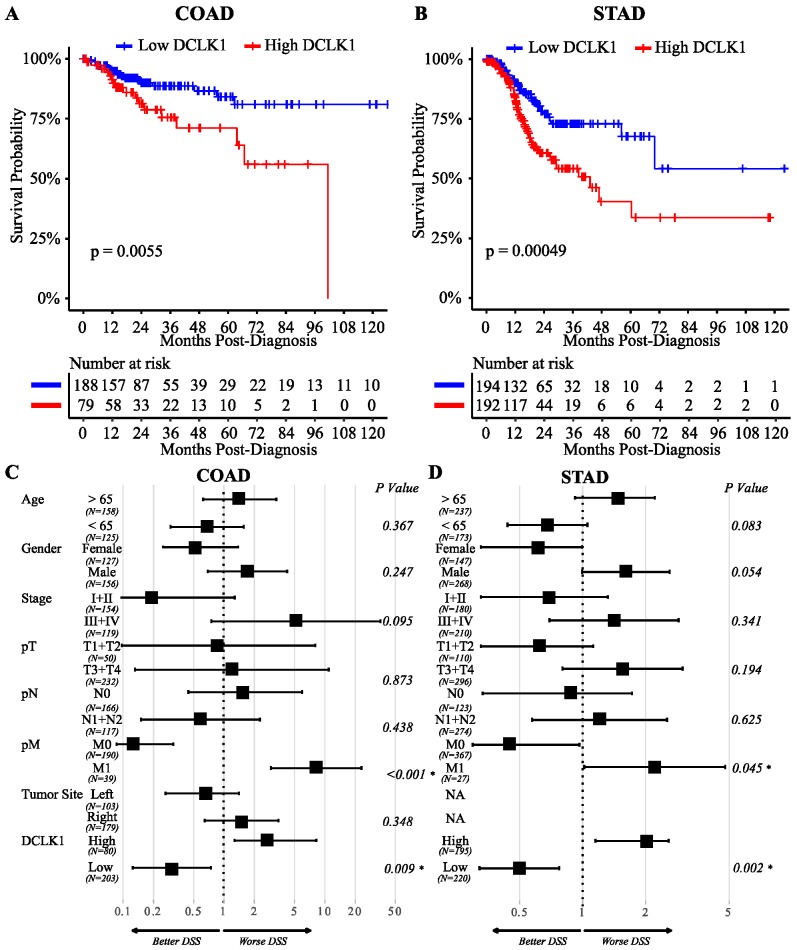
DCLK1 is an independent risk factor to predict disease-specific survival (DSS) of colon and gastric cancer. Lower DCLK1 mRNA expression significantly predicts shorter DSS in COAD (*n* = 283) (**A**) and STAD (*n* = 415) (**B**) based on Kaplan–Meier analysis. Multivariate Cox regression analysis of patients in COAD (**C**) and STAD (**D**) indicating that DCLK1 mRNA expression is an independent indicator of prognosis.

Previous reports indicate that DCLK1 is overexpressed in pancreatic, colorectal, and kidney cancers [41]. To explore the potential of using DCLK1 as a prognostic biomarker in GI cancers, we performed Kaplan–Meier and Cox regression analysis using the TCGA RNA-seq datasets [56,57]. High DCLK1 expression in tumor tissues predicted poor disease-specific survival (DSS) in both colon and stomach cancer patients (*p* < 0.006) (Figure 1A,B). In addition, high DCLK1 expression in the tumor tissues also predicted poor overall survival (OS; *p* = 0.021 for colon adenocarcinoma (COAD) and *p* = 0.0002 for stomach adenocarcinoma (STAD)), and progression-free survival (PFI) (*p* = 0.0086 for COAD and *p* < 0.0001 for STAD) (Figure S1A–D). By performing multivariate analysis to control for relevant clinical factors including age, gender, stage, T, N, M, and tumor location, we found that DCLK1 is an independent factor which can be used to predict poor DSS (*p* = 0.009, Figure 1C and Table S3) and PFI (*p* = 0.024, Table S4), but not OS (*p* = 0.18, Table S4) in CRC. In STAD, DCLK1 expression can be used as an independent factor to predict poor DSS (*p* = 0.002, Figure 1D and Table S3), OS (*p* = 0.008, Table S5), and PFI (*p* < 0.001, Table S5). These findings expand on previous findings demonstrating that tumor DCLK1 predicts survival in colon and stomach cancer [34,58,59] and suggest its independence as a prognostic biomarker.

### 2.2. DCLK1 Expression Levels Significantly Correlate with TME Immune and Stromal Scores

The tumor microenvironment and its interactions with the tumor epithelium are essential to cancer progression and metastasis. We used the well-established ESTIMATE algorithm to calculate immune and stromal proportions of COAD and STAD tumors (Figure S2A,B). Using Kaplan–Meier and Cox regression, we found that high immune score correlates with poor DSS in COAD (Figure S3A) and STAD (Figure S3B), in agreement with previous reports [60]. These results indicate that immune and stromal scores may be a useful indicator of colon and stomach cancer prognosis, but the underlying mechanisms need further study.

**Figure 2:**
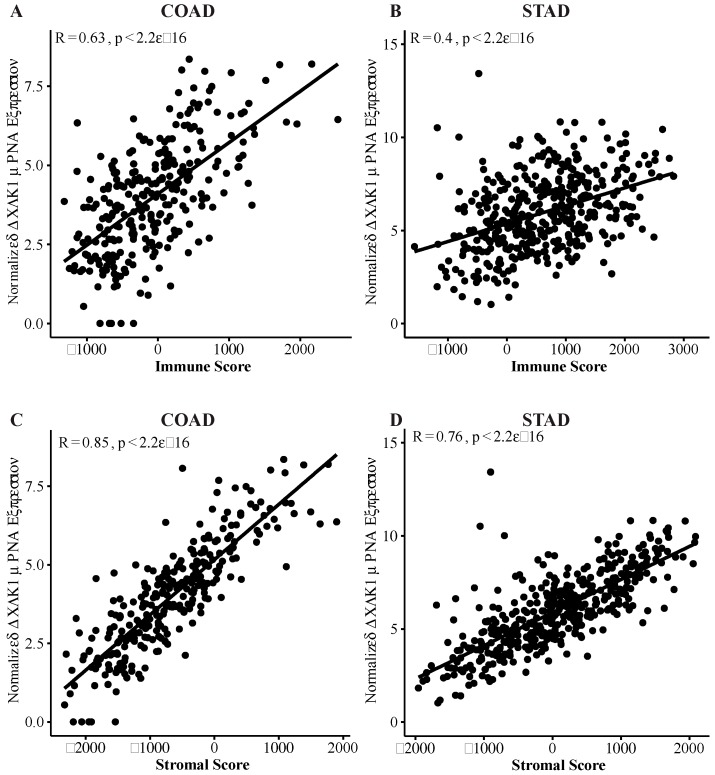
DCLK1 is correlated with an activated tumor microenvironment (TME) in COAD and STAD. DCLK1 mRNA expression is strongly associated with Immune Score and Stromal Score in COAD (**A** and **C**) and STAD (**B** and **D**) based on Pearson correlation analysis.

We found that tumor DCLK1 expression correlated with immune score in COAD and STAD (Pearson *r* = 0.63, *p* < 0.0001 and Pearson *r* = 0.4, *p* < 0.0001, respectively), and stromal score (Pearson *r* = 0.85, *p* < 0.0001 and Pearson *r* = 0.76, *p* < 0.0001, respectively) (Figure 2). Further analysis demonstrates that this correlation exists in a stage-independent fashion (Figure S4A) and STAD (Figure S4B). Together, this data suggests that the independent prognostic prediction potential of DCLK1 in colon and stomach cancer patients may be related to alterations in the TME.

### 2.3. DCLK1 Expression Level is Correlated with Various Immune Cell Subtypes in Both Colon and Stomach Cancer

**Figure 3:**
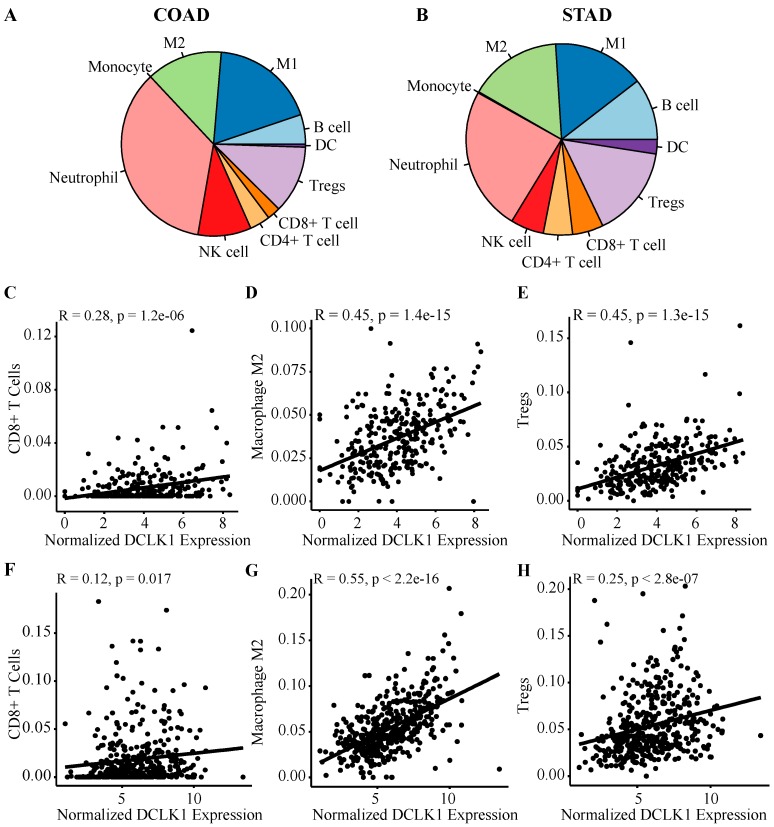
DCLK1 mRNA expression in tumor tissues is significantly correlated with immune infiltration. Estimated fraction of immune cells in tumor tissues of COAD (**A**) and STAD (**B**) was visualized by QuanTIseq analysis. Among all the immune cell types, macrophages (M1 + M2) represented the major population in COAD and STAD. Correlation analysis shows that DCLK1 expression is significantly correlated with CD8+ T cell (**C**), M2 macrophage (**D**), and Treg (**E**) in COAD. DCLK1 expression is also significantly correlated with M2 macrophage (**G**) and Treg (**H**) and shows a weak correlation with CD8+ T cells (**F**) in STAD.

To further study the relationship of DCLK1 to immune cell subtypes in the TME, we fragmented the composition of immune cells in tumor tissues of COAD and STAD using quanTIseq. Among immune cell types, macrophages (M1 + M2) accounted for about 32% and 31%, neutrophils accounted for approximately 24% and 35%, Treg accounted for 15% and 12%, and B cells accounted for 10% and 5% in COAD and STAD, respectively (Figure 3A,B). DCLK1 expression level in colon tumor tissues was positively correlated with infiltrating CD8+ T cells (*r* = 0.28, *p* < 0.0001), M2 macrophages (*r* = 0.45, *p* < 0.0001), Treg (*r* = 0.45, *p* < 0.0001) (Figure 3C–E), B cells (*r* = 0.42, *p* < 0.0001), and M1 macrophages (*r* = 0.24, *p* < 0.0001) (Figure S5A,D) in COAD. No significant correlation was found between DCLK1 expression and neutrophils, CD4+ T, dendritic, and NK cells (Figure S5B,C,E,F) in COAD. Similarly, it positively correlated with M2 macrophages (*r* = 0.55, *p* < 0.0001), Treg (*r* = 0.25, *p* < 0.0001) (Figure 3G,H), B cells (*r* = 0.3, *p* < 0.0001), CD4+ T cells (*r* = 0.33, *p* < 0.0001), and dendritic cells (*r* = 0.29, *p* < 0.0001) (Figure S5G,I,K) in STAD. No significant correlation was found between DCLK1 expression and CD8+ T cells (Figure 3F), neutrophils, M1 macrophages, and NK cells (Figure S5H,J,L). Among these immune cell types, DCLK1 expression had the strongest correlation with the M2 macrophage population (*r* = 0.45 and 0.55 for COAD and STAD, respectively). These findings suggest that DCLK1 may play a role in regulating TME composition, especially as it relates to immune infiltration in colon and gastric cancers.

### 2.4. DCLK1 Expression Level is Associated with TAM and M2 Macrophage Markers

**Figure 4:**
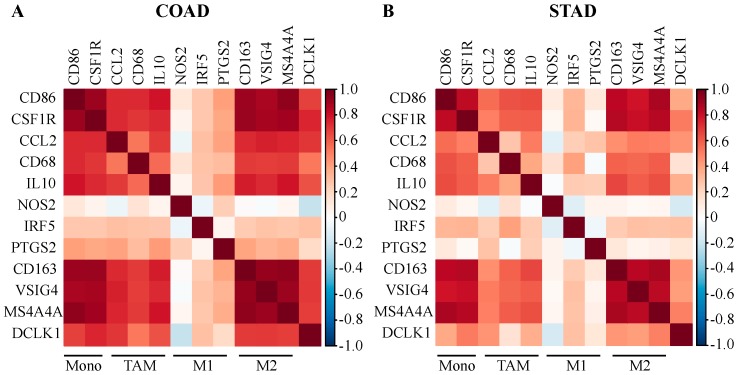
DCLK1 expression in tumor tissues correlates with macrophage polarization in COAD and STAD. Correlations analysis demonstrates that DCLK1 expression is significantly positively correlated with gene markers of monocytes (CD86 and CSF1R), TAMs (CCL2, CD68, and IL10), and M2 (CD163, VSIG4, and MS4A4A), but is not with gene markers of M1 (NOS2, IRF5, and PTGS2) in COAD (**A**) and STAD (**B**).

To further understand the relationship between DCLK1 and diverse infiltrating immune cell types, we analyzed the correlation between DCLK1 and signature genes for each immune cell type. We also analyzed the correlation with various functional T cell subtypes including Th1, Th2, Tfh, Th17, and Treg cells as well as T cell exhaustion. DCLK1 expression level was significantly correlated with CD8+ T cells, general T cells, B cells, monocytes, M2 macrophages, dendritic cells, neutrophils, Tfh, Treg, and T cell exhaustion in COAD and STAD (Table 1). The strongest correlations were with immune cell markers for Treg (FOXP3, CCR8, STAT5B, CD4, CD25, and TGFB1), M2 (CD163, VSIG4, and MS4A4A), and T cell exhaustion (PD-L1, CTLA4, LAG3, TIM-3, TIGIT, and BTLA), which suggests that high DCLK1 expression in an M2-enriched TME may restrict antitumor immunity [31,61]. We also found that the DCLK1 expression level has strong positive correlations with markers of monocytes, TAMs, and M2 macrophages in COAD (Figure 4A) and STAD (Figure 4B). These findings suggest that DCLK1 may regulate macrophage polarization in COAD and STAD.

### 2.5. DCLK1 Correlates with Evasion of Antitumor Immunity, TAM Activation, and Inhibition of CD8+ T Cells

**Figure 5:**
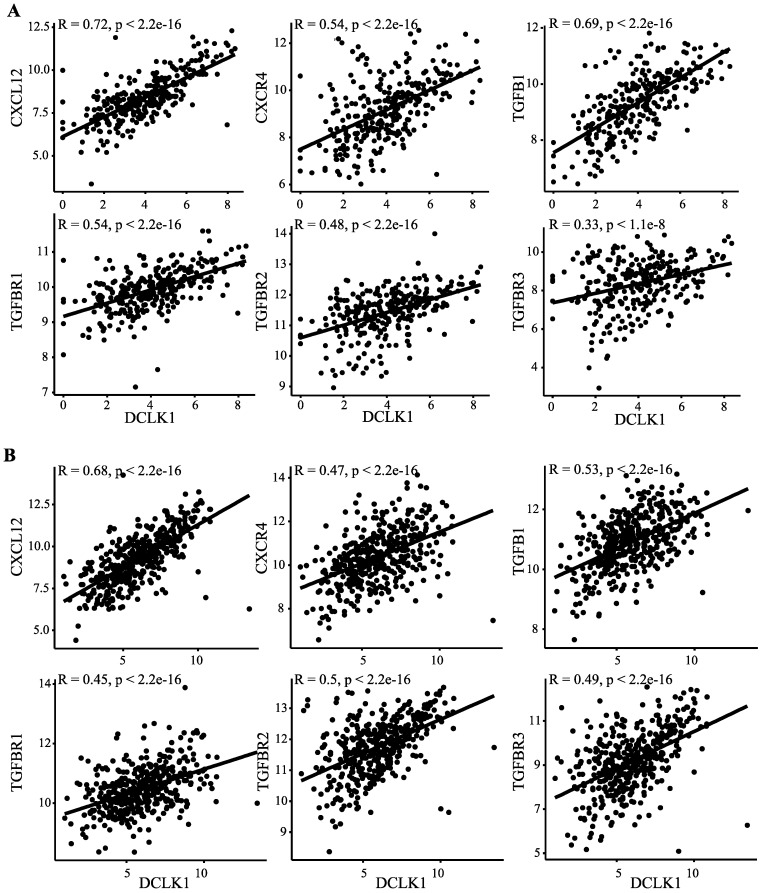
DCLK1 expression is positively associated with CD8+ T cell inhibitors induced by TAMs. Correlation analysis suggests that DCLK1 expression is significantly positively correlated with TGF-B1 and CXCL12 and their specific receptors TGF-BR1, TGF-BR2, and TGF-BR3, and CXCR4, respectively, in COAD (**A**) and STAD (**B**).

**Figure 6:**
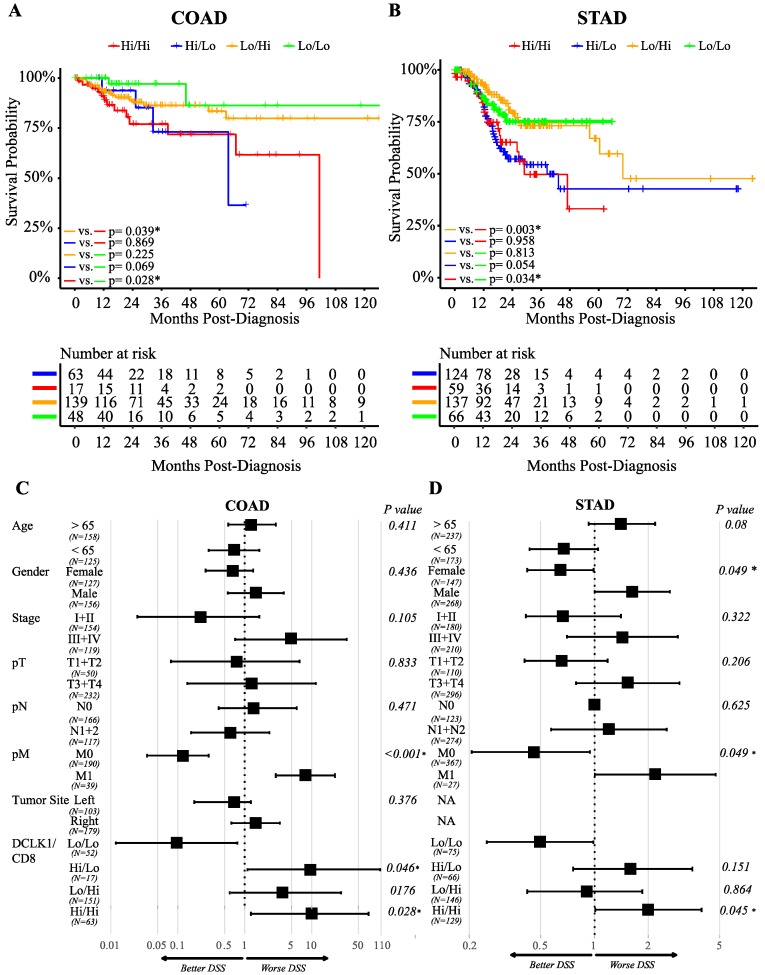
DCLK1/CD8 status predicts DSS of colon and stomach cancer patients. Kaplan–Meier analysis of composite DCLK1 and CD8 status based on mRNA expression in COAD (**A**) and STAD (**B**). DCLK1Lo/CD8Lo (green line) patients presented with better survival times than DCLK1Hi/ CD8Hi (red line) for COAD and STAD. DSS times of COAD and STAD patients with lower DCLK and higher CD8 (yellow line) were better than higher DCLK1 and higher CD8 (red line). Cox regression analysis of patients in COAD (**C**) and STAD (**D**) indicated that combined high DCLK1 and high CD8 mRNA expression is an independent indicator of prognosis. DCLK1 Lo/CD8Lo was used as the control group for all statistical comparisons.

Recent studies show that M2-like TAMs inhibit CD8+ T cell infiltration through inducing TGFB1 and CXCL12 [25,26,62]. We found that DCLK1 expression significantly correlates with both TGFB1 and CXCL12 and their receptors TGFBR1, TGFBR2, and TGFBR3, and CXCR4, respectively, in colon and stomach cancer patients (Figure 5A,B). These results indicate that DCLK1 may contribute to TAM development by preventing CD8+ T cell infiltration. To assess the impact of this activity on patient outcomes, we divided COAD and STAD patients into four groups: DCLK1^Hi^/CD8+^Hi^, DCLK1^Hi^/CD8+^Lo^, DCLK1^Lo^/CD8+^Hi^, and DCLK1^Lo^/CD8+^Lo^ and performed Kaplan–Meier analysis. DCLK1^Lo^/CD8+^Lo^ tumors (green line) indicated better DSS compared to DCLK1^Hi^/CD8+^Lo^ tumors (blue line), and DCLK1^Lo^/CD8+^Hi^ tumors (yellow line) indicated better DSS than DCLK1^Hi^/CD8+^Hi^ tumors (red line) in COAD (Figure 6A) and STAD (Figure 6B). Controlling for relevant clinical factors including age, gender, stage, T, N, M, and tumor location demonstrated that DCLK1^Hi^/CD8+^Hi^ status is an independent factor predictor of DSS in COAD and STAD (*p* = 0.028 for COAD and *p* = 0.045 for STAD, Figure 6C,D). These results suggest that further studies should be performed to determine DCLK1’s role in activating TAMs and inducing stromal cell secretion of CD8+ T cell inhibitors TGFB1 and CXCL12 to reduce the CD8+ T cell infiltration in the TME.

### 2.6. DCLK1 Predicts the Survival of Patients with Left-Sided Colon Cancer

**Figure 7:**
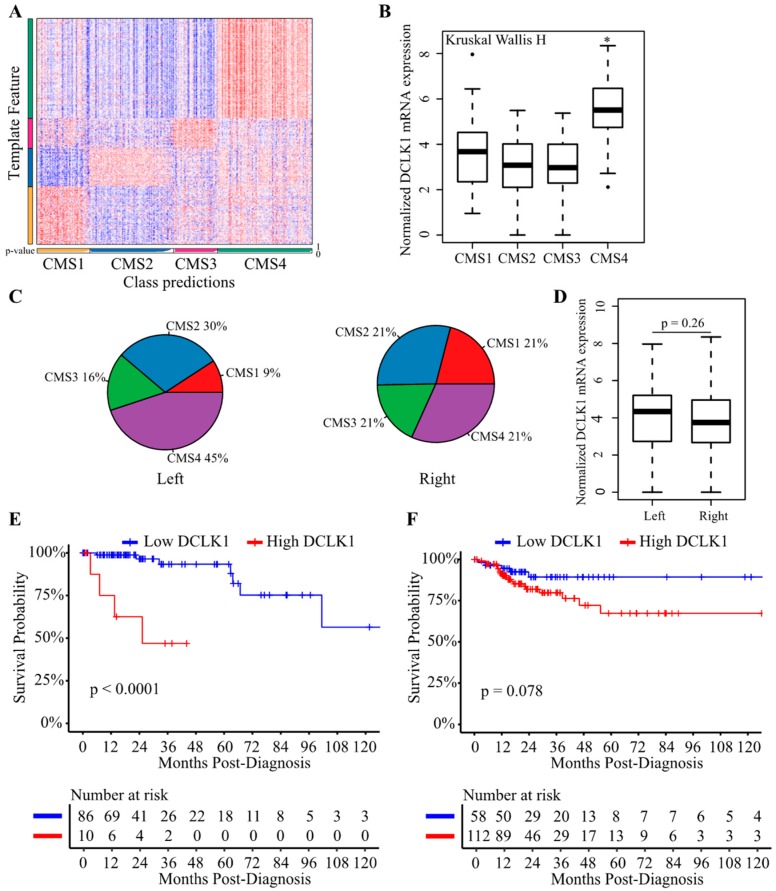
DCLK1 predicts DSS in the patients with left-sided colon cancer. (**A**) CMScaller performance on the test set of COAD from TCGA (*n* = 283). Heatmap represents the relative expression levels of subtype marker genes (vertical bar) with classifications indicated below (horizontal bar, white indicates prediction confidence *p*-values). (**B**) DCLK1 expression level was significantly increased in CMS4 colon cancers. (**C**) Colon cancer initiated in the left colon (descending colon, sigmoid colon, and rectum) was more likely to present as CMS4 compared with those initiated in the right colon (caecum, ascending colon, and transverse colon). (**D**) DCLK1 mRNA level showed no significant difference between left-sided and right-sided colon cancers. Higher DCLK1 expression predicts worse DSS in patients with cancers initiated in the left colon (**E**) but not in the right colon (**F**) based on Kaplan–Meier analysis.

Consensus molecular subtypes (CMS) can be used to divide CRCs into four biologically distinct and clinically relevant subtypes: CMS1 (MSI-like), CMS2 (canonical), CMS3 (metabolic), and CMS4 (mesenchymal) [63,64]. Recent studies also highlight left-sided (descending colon, sigmoid colon, and rectum) and right-sided (caecum, ascending colon, and transverse colon) colon cancers as having distinct molecular features which result in different outcomes and drug responses [65]. To determine the DCLK1 expression in these contexts, we performed the CMS analysis of COAD dataset (Figure 7A). DCLK1 expression was upregulated specifically in the CMS4 which has the worst outcomes among the four subtypes (Figure 7B) [66]. In addition, we analyzed the CMS subtypes of left-sided and right-sided colon cancers, and found that left-sided colon cancer was more likely to be CMS4 (Figure 7C). DCLK1 expression was similar in left-sided and right-sided colon cancer (Figure 7D). To assess the specificity of DCLK1 as a prognostic marker in these contexts, Kaplan–Meier analysis was performed and showed that higher DCLK1 expression predicted worse DSS in left- but not right-sided colon cancer (Figure 7E,F). These results further extended our knowledge about the functional role of DCLK1 in colon cancer prognosis and may contribute to developing a specific treatment strategy to treat left-sided colon cancers.

Stomach cancer can be classified as cardia, fundus/body, and antrum based on tumor location and MSS, MSI-Low, and MSI-High according to molecular features [67,68]. DCLK1 mRNA expression was upregulated in tumors originating anatomically in the area of the antrum compared with cardia and fundus/body (Figure S6A) and in MSS subtype compared with MSI-Low and MSI-High subtype (Figure S6B). In addition, Kaplan–Meier analyses were performed and showed that higher DCLK1 expression predicted worse DSS in every subtype stomach cancer except the MSI-Low subtype (Figure S6C–H).

## 3. Discussion

Despite advances in the understanding of GI cancer biology and in surgery and immunotherapy in recent years, little impact has been made on the mortality associated with advanced GI cancers [15,16,66]. Therefore, there is an unmet need to find new immune-related therapeutic approaches against GI cancer. Previous studies reported that DCLK1 level in colorectal, gastric, pancreatic, breast, and esophageal cancers and clear renal cell carcinoma is higher in tumor tissues compared with adjacent tissues [37,39,69,70,71,72], and it can predict poor survival in kidney and pancreatic cancers [35,41]. Our findings again confirm that DCLK1 can be used as a prognostic biomarker for colon and gastric cancer, and extend understanding of this potential to clinically relevant subtypes.

The TME, consisting of numerous cell types including blood, immune, and stromal cells and extracellular matrix, is essential to tumor initiation and progression as well as the development of immune escape mechanisms [25]. Stromal and immune infiltration have previously been associated with clinical outcomes of patients and chemotherapy resistance using multiple cohorts [60]. Similarly, our results show that stromal and immune infiltration are associated with clinical outcomes in colon and stomach cancer (Figure S3A,B). In addition, we identified DCLK1 mRNA expression as positively correlated with stromal and immune scoring. DCLK1 is a marker of gastrointestinal tuft cells which drive the type II immune response to inflammatory injury and have been reported to play an important role in cancer initiation and development in various cancers [14,33]. These correlations suggest that DCLK1-based prediction of outcome in colon and stomach cancers may be associated with stromal and immune cells in the TME.

Immune cell infiltration in the TME may affect tumor cell survival, metastasis, and therapy resistance [25,30,31,73]. Our current results demonstrate that DCLK1 expression is correlated with infiltration of multiple immune cell types in colon and gastric cancer. Notably, there is a weak positive relationship between DCLK1 expression levels and infiltration levels of CD8+ T cells, and significant positive correlations between infiltration levels of M2 macrophages and Treg (Figure 3C,D and Figure 4). Correlation between DCLK1 expression and the markers of immune cells implicates DCLK1 in regulating antitumor immunity in COAD and STAD. Markers of M1 macrophages such as NOS2 and PTGS2 showed no correlation and IRF5 showed weak correlation with DCLK1, whereas M2 macrophages markers such as CD163, VSIG4, and MS4A4A showed strong correlation. Therefore, it is tempting to speculate that DCLK1 or the DCLK1+ cell may regulate polarization of tumor-associated macrophages (TAMs). Furthermore, our results indicate a relationship between DCLK1 and activation of Treg and T cell exhaustion. Specifically, higher DCLK1 expression is associated with increased Treg (FOXP3, CCR8, STAT5B, TGF-B1, CD25, and CD4) and T cell exhaustion markers (PD-1, CTLA4, LAG3, TIM-3, TIGIT, and BTLA) in COAD and STAD (Table 1). Among these markers, TGF-B1, CXCL12, and TIM-3 are crucial to the induction of T cell exhaustion and show the strongest correlation with DCLK1 [73]. Additional significant associations can be found between DCLK1 expression and B cell and DC (Figure S5A,G,K) and several markers of B cell, DC, and T helper cells (Th1, Th2, Tfh, and Th17) in both COAD and STAD. Despite DCLK1 expression showing moderate correlation with Th1-specific transcription factors (T-bet and STAT4), which are known to support Th1 cell cytokine secretion to restrict cancers [74,75,76,77,78], DCLK1 showed little correlation with direct tumor-inhibiting factors such as IFN-gamma, TNF-alpha, and IL12 in COAD and STAD (Table 1) [79,80]. Similarly, for DC and B cell markers, DCLK1 expression indeed positively correlated with CD19, CD79A (B cell markers), and activated DC cell markers (HLA+ class II, CD1b, and CD1c), but several studies indicate that B cells play a negative role in tumor immunity through cytokines IL10 and IL35, which showed positive correlation with DCLK1 expression [81,82]. Moreover, the effectors of DC cells, IL12 and IL1, showed a lack of or negative correlation with DCLK1 expression [83,84,85]. Together, these findings suggest that DCLK1 may play an important role in recruitment and regulation of immune infiltrating cells in COAD and STAD and mechanistic studies are warranted.

CD8+ cytotoxic T cells are a key effector in tumor cell eradication. Higher CD8+ T cell infiltration has been correlated with better clinical outcomes in many cancers [25,73]. Our findings show that low DCLK1 expression correlated with high or low CD8 + T cells predicts better survival in colon and stomach cancer patients, suggesting DCLK1 has the potential to be an adjuvant target to promote sensitivity in immunotherapy-resistant patients. Additionally, it is widely reported that exhausted CD8+ T cells also express CD8A but lose the ability to eradicate the tumor cell [86,87]. This is hypothesized to be one significant reason why immune blockade increases the function of exhausted CD8+ T cells [88,89]. Taken together, our findings that DCLK1 is positively correlated with CD8+ T cells, combined with our findings that DSS in patients with low DCLK1 expression and high CD8+ T cells showed no significant difference compared with low DCLK1 expression and low CD8+ T cells, might indicate that DCLK1 can be used to predict the effect of immunotherapies aimed at restoring function in exhausted CD8 T cells.

Colon cancer is a frequently lethal disease with heterogenous outcomes and drug responses resulting from varying molecular characteristics [64]. Important findings show that left-sided colon cancer has worse clinical outcomes than right-sided colon cancer [65]. Our study demonstrated significant overlap between left-sided cancer and the CMS4 population which has the worst clinical outcomes among the four CMS molecular subtypes of colon cancer. Though DCLK1 mRNA expression showed no significant difference between left-sided cancer and right-sided cancer, higher DCLK1 expression predicted poor outcomes in left-sided cancer patients (Figure 7). These results extend our knowledge about left-sided colon cancer resistance and further suggest that DCLK1 has potential as a specific target in left-sided colon cancers. Additionally, stomach cancer can be classified based on anatomic location and molecular features. Our findings demonstrated that DCLK1 can almost predict the survival of all the specific subtypes for the first time (Figure S6), suggesting DCLK1 plays an important role in stomach cancer and more sophisticated classification to predict different prognoses deserves further study.

## 4. Materials and Methods

### 4.1. TCGA Colon and Stomach Cancer Dataset

The RNA-seq datasets from the February 2015 data runs for colon adenocarcinoma (COAD) and stomach adenocarcinoma (STAD) were downloaded from the University of California, Santa Cruz (UCSC) Cancer Genome Browser.

### 4.2. Clinical Patient Characteristics

Only publicly available, deidentified data were accessed from TCGA for the analyses reported here. Basic characteristics for the patients used in the survival analyses are provided in Table S1.

### 4.3. Estimation of Tumor Cellular Components and CRC Subtypes

The stromal and immune scores were calculated using the ESTIMATE packages in R [90,91]. The expression profiles of 141 stroma-related genes and 141 immune-related genes (provided in Table S2) were analyzed to obtain stromal score and immune score, respectively. By running ESTIMATE on TCGA RNA-seq data, the stromal and immune score of each sample can be estimated as previously described [90,91,92]. We quantified the proportion of cells that belonged to each of 10 immune cell types (B cells, M1 macrophages, M2 macrophages, monocytes, neutrophils, NK cells, CD4+ T cells, CD8+ T cells, regulatory T cells, and dendritic cells) using the quanTIseq package in R [93]. CMS classifications of COAD were performed using the CMSCaller package in R [64].

### 4.4. Visualization of RNA Sequencing Dataset

All the figures in the current study were generated in R v3.6.0. The ggsurvplot and ggforest functions from the “survminer” package were used to generate Kaplan–Meier and forest plots, respectively. The base pie function was used to display estimated fraction of immune cells in tumor tissues. The ggscatter function from the “ggpubr” package was used to visualize binary correlations. The corrplot function from the “corrplot” package was used to determine correlations between DCLK1 mRNA expression and gene markers of macrophages. The base boxplot function was used to generate mRNA expression plots of DCLK1 in various conditions.

### 4.5. Statistical Analysis

Basic statistical analyses were performed in R v3.6.0 and IBM SPSS 25.0. The Wilcox test and Kruskal–Wallis H test were used to determine significance for nonparametric data; *p* value < 0.05 was considered statistically significant except for correlation analysis. Pearson correlations were calculated with the cor.test package in R and correlation plots were prepared with ggpubr and corrplot packages in R. For correlations only *p* values < 0.0001 were considered statistically significant, and the strength of the correlation was described as follows: 0.00–0.19 “very weak”, 0.20–0.39 “weak”, 0.40–0.59 “moderate”, 0.60–0.79 “strong”, 0.80–1.0 “very strong” [94]. Kaplan–Meier and Cox regression analyses were performed and visualized using the survival and survminer packages in R. The optimal cutpoint for these analyses was generated by the function “surv_cutpoint” which originates in the “maxstat” R package [95,96].

## 5. Conclusions

In summary, in colon and gastric cancers, increased DCLK1 expression correlates with poor prognosis and increased infiltration of M2 macrophages and Treg. DCLK1 expression potentially contributes to regulation of tumor-associated macrophages (TAMs) and Treg, and may thereby increase CD8+ T cell inhibition and induce T cell exhaustion. These functional roles in the TME may be specific to left-sided subtypes in colon cancer and DCLK1 may have potential as a prognostic biomarker and adjuvant target to promote immunotherapy sensitivity to improve outcomes in colon and gastric cancer patients.

## Figures and Tables

**Table 1:** Correlation analysis between DCLK1 and related genes and markers of immune cells.

Description	Gene Markers	COAD				STAD			
		Cor	95%CI		*p*	Cor	95%CI		*p*
CD8+ T cell	CD8A	0.412	0.311	0.503	***	0.328	0.239	0.411	***
	CD8B	0.253	0.141	0.358	***	0.187	0.092	0.278	0.0001
T cell (general)	CD3D	0.415	0.315	0.507	***	0.258	0.166	0.346	***
	CD3E	0.512	0.421	0.593	***	0.303	0.213	0.388	***
	CD2	0.476	0.381	0.561	***	0.320	0.231	0.404	***
B cell	CD19	0.477	0.382	0.562	***	0.372	0.286	0.452	***
	CD79A	0.568	0.484	0.642	***	0.396	0.312	0.474	***
	IL35	0.559	0.474	0.634	***	0.355	0.268	0.436	***
Monocyte	CD86	0.680	0.612	0.738	***	0.379	0.293	0.458	***
	CD115(CSF1R)	0.748	0.692	0.795	***	0.522	0.448	0.589	***
	CCL2	0.704	0.640	0.758	***	0.445	0.364	0.519	***
	CD68	0.525	0.436	0.604	***	0.160	0.064	0.252	0.001
	IL10	0.633	0.558	0.698	***	0.363	0.276	0.444	***
M1 Macrophage	INOS(NOS2)	−0.207	−0.315	−0.093	0.0004	−0.147	−0.240	−0.052	0.003
	IRF5	0.292	0.182	0.395	***	0.297	0.207	0.382	***
	COX2(PTGS2)	0.186	0.071	0.295	0.001	0.135	0.039	0.228	0.006
M2 Macrophage	CD163	0.692	0.627	0.748	***	0.437	0.356	0.512	***
	VSIG4	0.695	0.630	0.751	***	0.416	0.333	0.492	***
	MS4A4A	0.690	0.624	0.746	***	0.502	0.426	0.570	***
Neutrophil	CD66b(CEACAM8)	−0.129	−0.242	−0.013	0.029	−0.008	−0.104	0.089	0.875
	CD11b(ITGAM)	0.725	0.665	0.776	***	0.475	0.397	0.546	***
	CCR7	0.585	0.503	0.657	***	0.473	0.395	0.545	***
Natural killer cell	KIR2DL1	0.196	0.082	0.305	0.0008	0.181	0.086	0.272	0.0002
	KIR2DL3	0.275	0.164	0.379	***	0.105	0.009	0.199	0.032
	KIR2DL4	0.105	−0.011	0.218	0.076	−0.069	−0.164	0.027	0.160
	KIR3DL1	0.210	0.097	0.319	0.0003	0.181	0.086	0.273	0.0002
	KIR3DL2	0.281	0.171	0.385	***	0.138	0.042	0.231	0.032
	KIR3DL3	0.040	−0.076	0.155	0.500	−0.051	−0.146	0.046	0.302
	KIR2DS4	0.187	0.073	0.297	0.0014	0.076	−0.021	0.171	0.124
Dendritic cell	HLA-DPB1	0.648	0.576	0.711	***	0.311	0.222	0.396	***
	HLA-DQB1	0.386	0.283	0.480	***	0.142	0.047	0.235	0.004
	HLA-DRA	0.506	0.414	0.587	***	0.211	0.117	0.301	***
	HLA-DPA1	0.583	0.501	0.655	***	0.268	0.176	0.355	***
	BDCA-1(CD1C)	0.668	0.598	0.728	***	0.567	0.497	0.629	***
Th1	BDCA-1(NRP1)	0.760	0.707	0.805	***	0.635	0.574	0.689	***
	CD11c(ITGAX)	0.635	0.560	0.699	***	0.405	0.321	0.482	***
	CD11b (ITGAM)	0.725	0.655	0.776	***	0.475	0.397	0.546	***
	IL12A	0.046	−0.07	0.161	0.437	0.192	0.098	0.283	***
	IL1B	−0.228	−0.318	−0.135	***	0.068	−0.048	0.183	0.246
	T-bet (TBX21)	0.483	0.388	0.567	***	0.301	0.211	0.386	***
	STAT4	0.530	0.442	0.609	***	0.396	0.311	0.474	***
	STAT1	0.374	0.270	0.470	***	0.004	−0.092	0.100	0.933
	IFN-gamma (IFNG)	0.158	0.043	0.269	0.007	−0.042	−0.137	0.055	0.398
	TNF-alpha (TNF)	0.339	0.232	0.438	***	0.011	−0.085	0.107	0.820
Th2	GATA3	0.628	0.552	0.693	***	0.424	0.342	0.500	***
	STAT6	−0.010	−0.126	0.106	0.860	0.163	0.067	0.255	0.0009
	STAT5A	0.393	0.290	0.487	***	0.421	0.339	0.497	***
	IL13	0.259	0.147	0.364	***	0.146	0.050	0.239	0.003
Tfh	BCL6	0.536	0.448	0.614	***	0.456	0.376	0.529	***
Th17	IL21	0.319	0.211	0.419	***	0.121	0.025	0.215	0.013
	STAT3	0.339	0.232	0.438	***	0.321	0.232	0.405	***
Treg	IL17A	−0.208	−0.316	−0.094	0.0003	−0.308	−0.393	−0.218	***
	FOXP3	0.615	0.537	0.682	***	0.241	0.148	0.329	***
T cell exhaustion	CCR8	0.628	0.552	0.693	***	0.353	0.266	0.435	***
	STAT5B	0.460	0.363	0.547	***	0.635	0.573	0.689	***
	TGFbeta (TGFB1)	0.692	0.626	0.748	***	0.530	0.457	0.596	***
	CD25 (IL2RA)	0.575	0.491	0.648	***	0.292	0.201	0.377	***
	CD4	0.708	0.646	0.762	***	0.424	0.342	0.500	***
	PD-1(PDCD1)	0.435	0.336	0.524	***	0.201	0.107	0.291	***
	CTLA4	0.478	0.383	0.563	***	0.106	0.010	0.200	0.031
	LAG3	0.341	0.234	0.439	***	0.140	0.045	0.233	0.004
	TIM-3(HAVCR2)	0.666	0.596	0.726	***	0.362	0.275	0.443	***
	BTLA	0.556	0.471	0.632	***	0.436	0.354	0.511	***
	TIGIT	0.541	0.453	0.618	***	0.344	0.256	0.426	***

*** *p* < 0.0001.

## Supplementary Materials

The following are available online at https://www.mdpi.com/2072-6694/12/2/274/s1, Figure S1: DCLK1 expression predicts clinical outcome of COAD and STAD. Figure S2: Histogram presentation of immune and stromal scores for COAD (A) and STAD (B). Figure S3: Immune score and stromal score predict DSS of COAD and STAD. Figure S4: DCLK1 expression correlates with immune and stromal score in a stage-independent fashion in COAD and STAD. Figure S5: DCLK1 mRNA expression in tumor tissues is significantly correlated with immune infiltration. Figure S6: DCLK1 mRNA expression predicts DSS in the patients of every subtype of STAD. Table S1: Clinical patient characteristics for the colon and stomach cancer. Table S2: Gene list of immune and stromal signatures. Table S3: Fisher test of DCLK1 expression in COAD and STAD. Table S4: Univariate analysis and multivariate analysis of OS and PFI among COAD according to clinical characteristic and DCLK1 expression. Table S5: Univariate analyses and multivariate analyses of OS and PFI among STAD according to clinical characteristic and DCLK1 expression.
