# Supplementary material for: Cancer Stem Cell Marker DCLK1 Correlates with Tumorigenic Immune Infiltrates in the Colon and Gastric Adenocarcinoma Microenvironments
Source: Cancers (Basel). 2020 Jan 22;12(2):274. doi: 10.3390/cancers12020274 (PMC7073156; doi:10.3390/cancers12020274)
Supplement: Supplementary file 1 [file cancers-12-00274-s001.pdf]

Supplementary Materials: Cancer Stem Cell Marker DCLK1 Correlates with Tumorigenic Immune Infiltrates in the Colon and Gastric Adenocarcinoma Microenvironments

Xiangyan Wu, Dongfeng Qu, Nathaniel Weygant, Jun Peng and Courtney W. Houchen

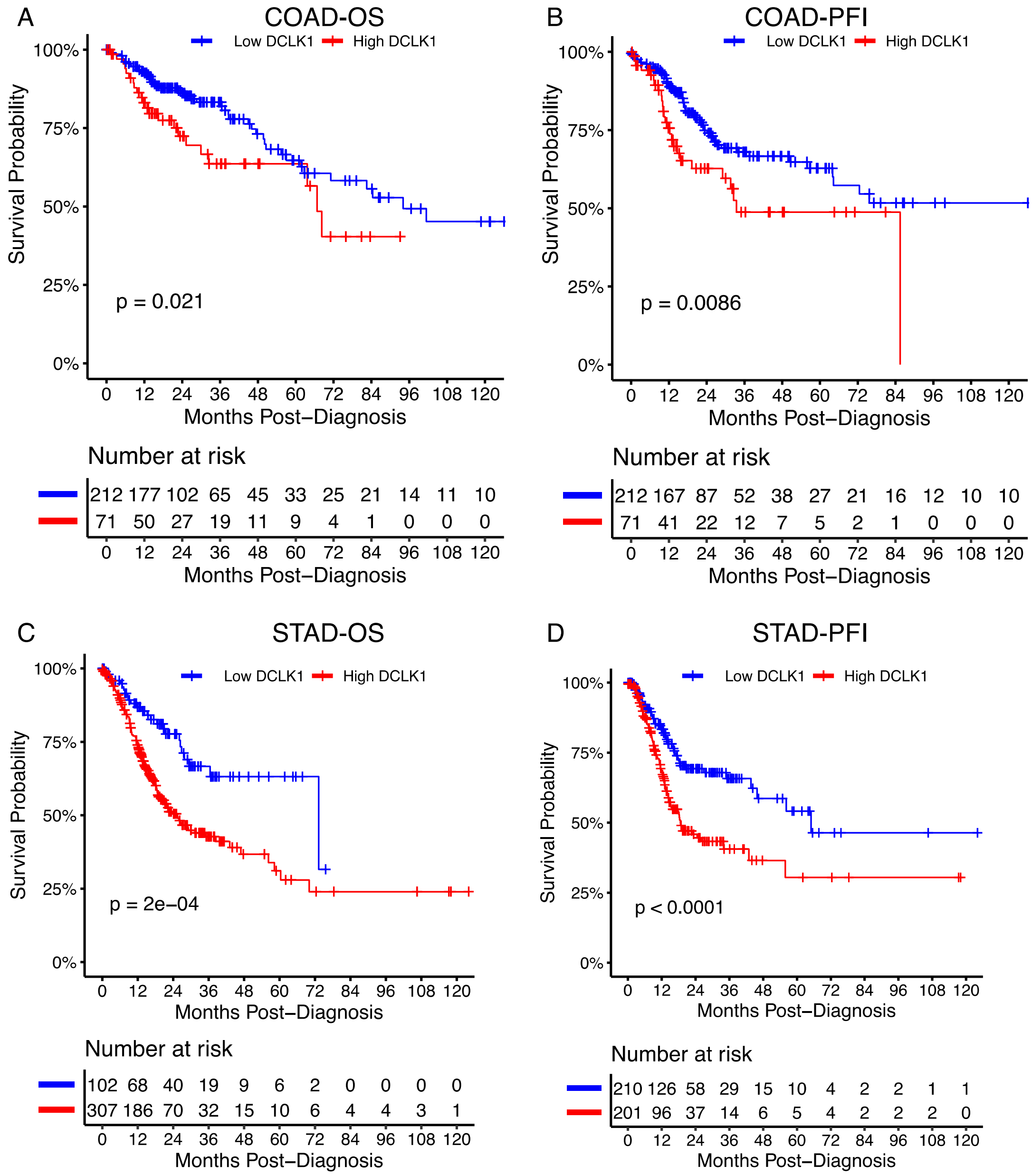

Figure S1. DCLK1 expression predicts clinical outcome of COAD and STAD. Kaplan-Meier analysis of DCLK1 mRNA expression in COAD (n = 283) and STAD (n = 415) demonstrating that high DCLK1 expression in tumor tissues predicts significantly shorter overall survival (OS) (**A**,**C**) and progression-free survival (PFI) (**B**,**D**).

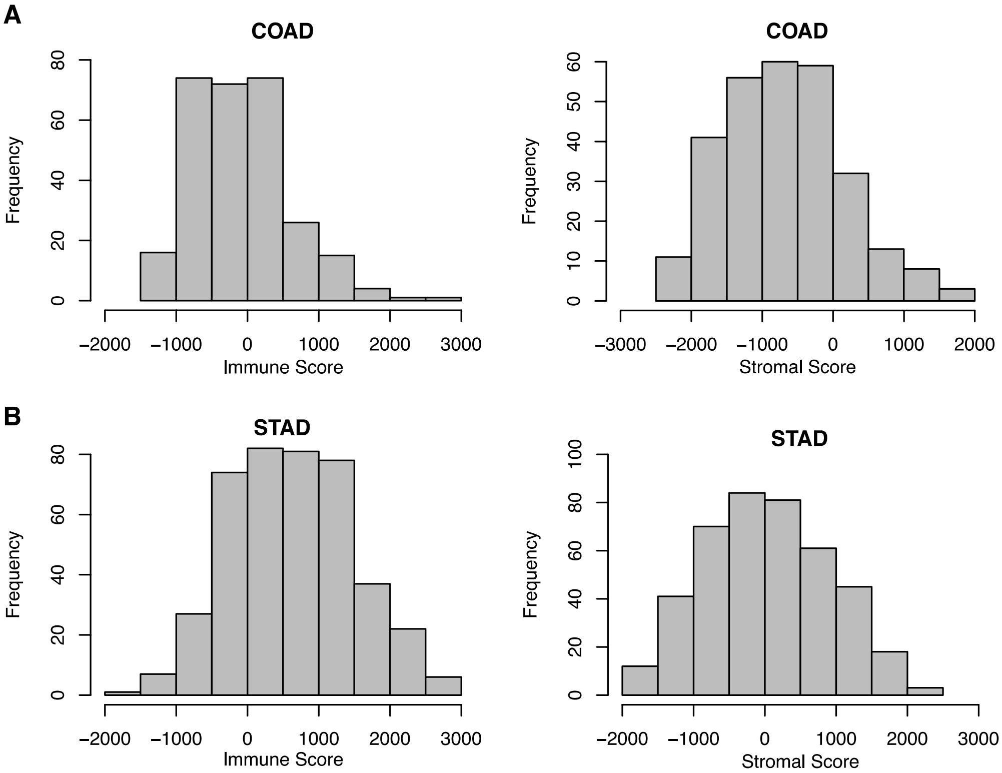

Figure S2. Histogram presentation of immune and stromal scores for COAD (**A**) and STAD (**B**).

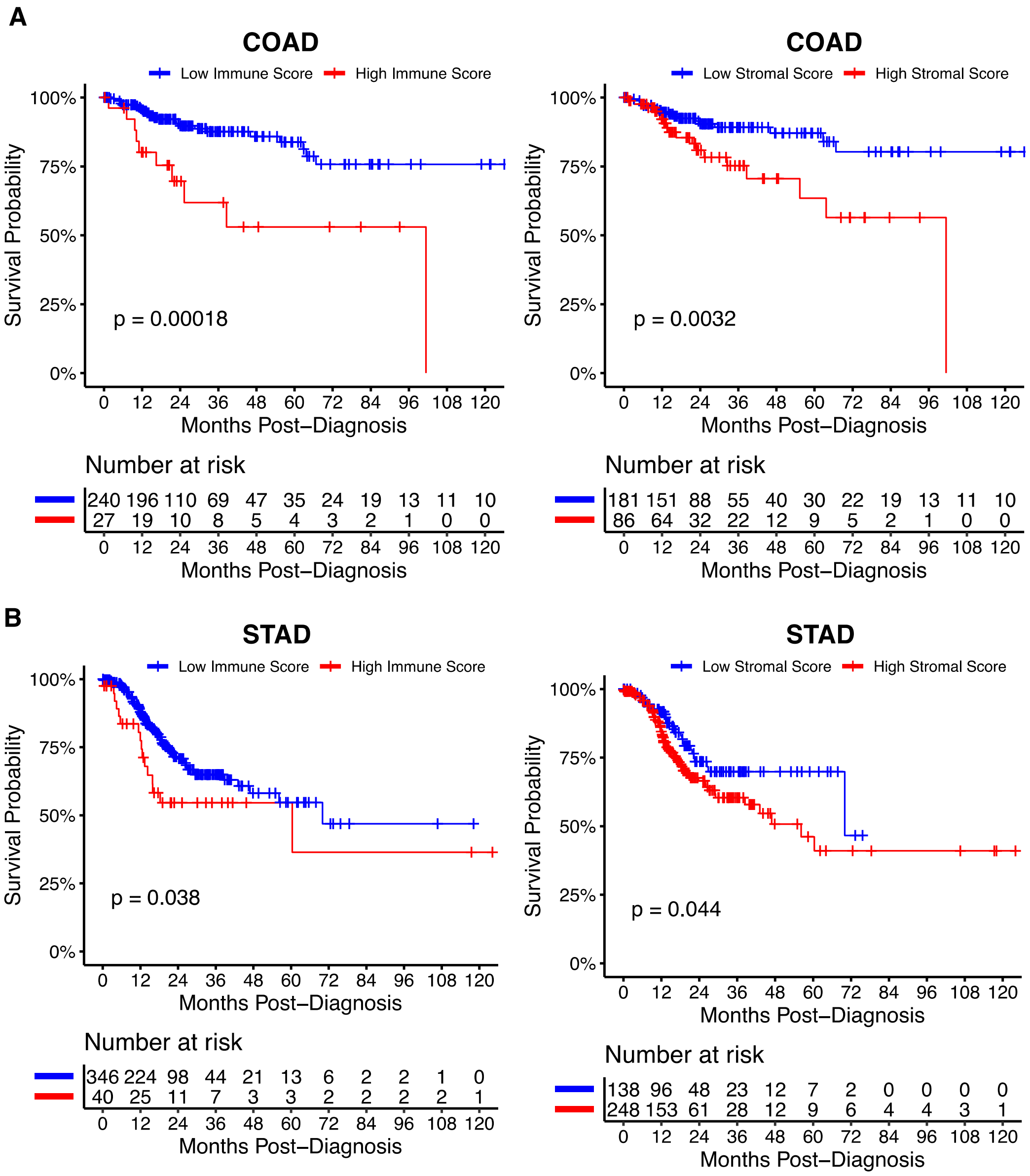

Figure S3. Immune score and stromal score predict DSS of COAD and STAD. Kaplan-Meier analysis of immune and stromal score in COAD and STAD indicating that high immune scores and high stromal score predict significantly shorter DSS in COAD (**A**) and STAD (**B**).

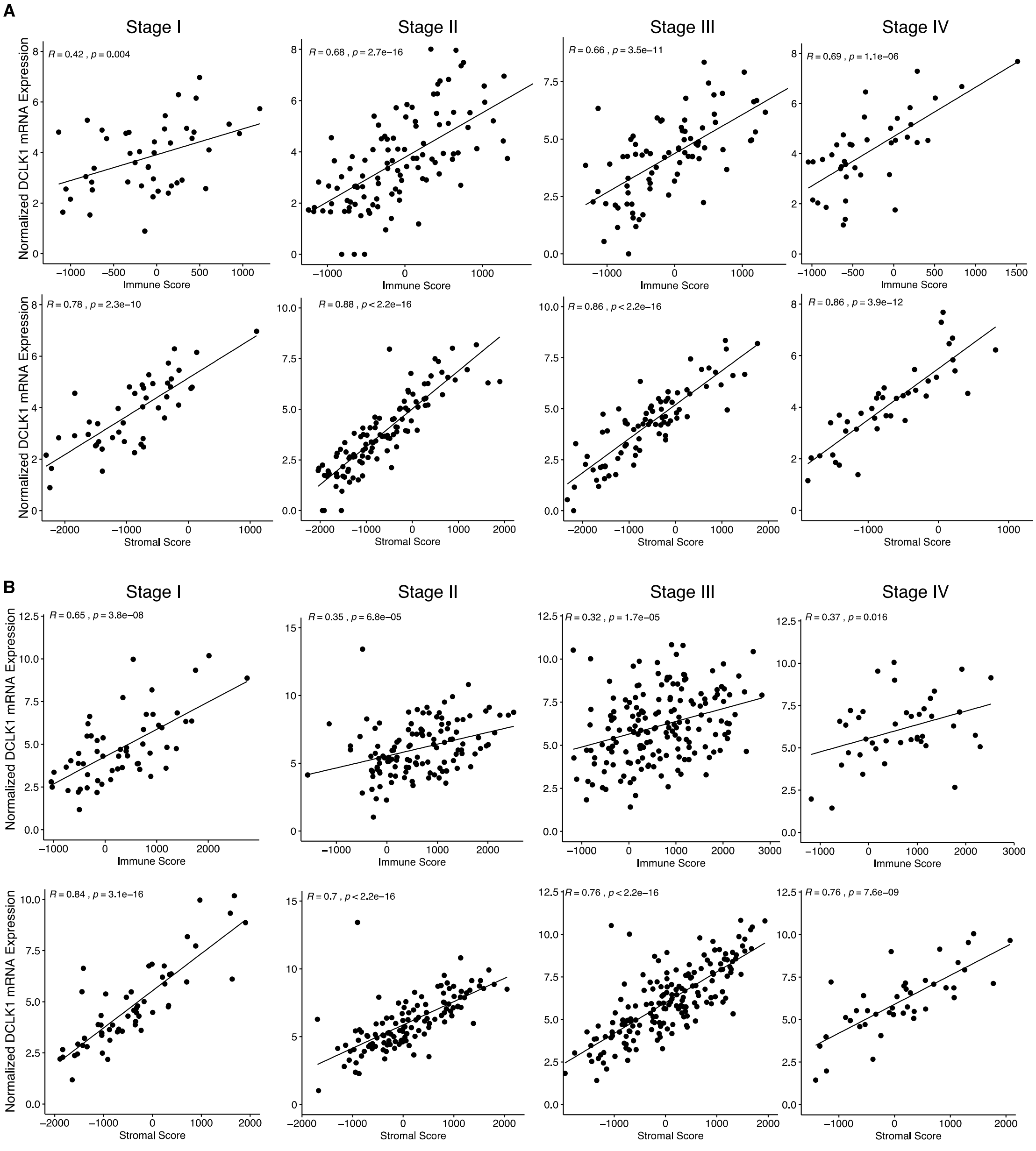

Figure S4. DCLK1 expression correlates with immune and stromal score in a stage-independent fashion in COAD and STAD. Correlations analysis demonstrates that DCLK1 expression is significantly positively correlated with immune and stromal scores in every disease stage of COAD (**A**) and STAD (**B**).

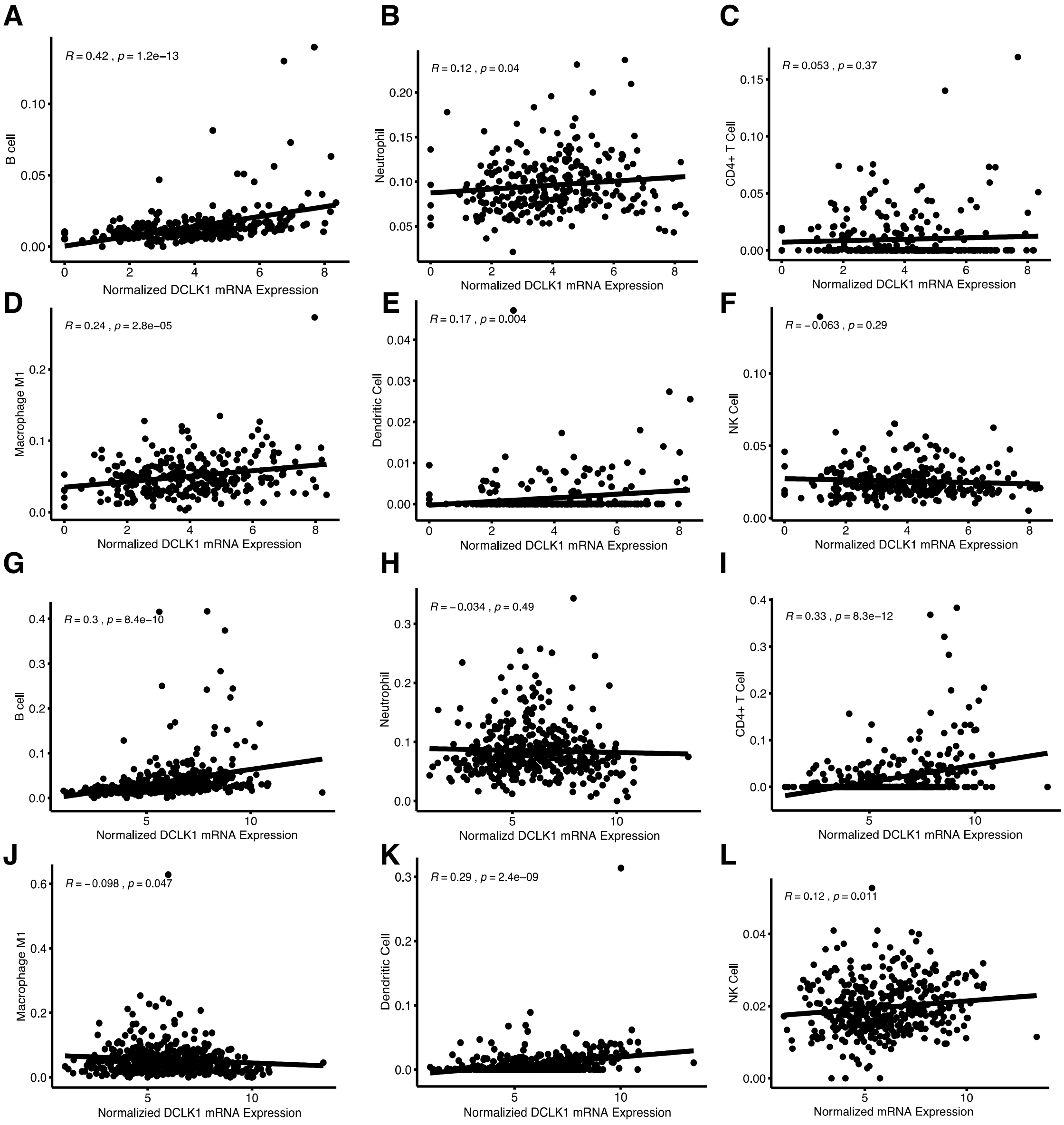

Figure S5. DCLK1 mRNA expression in tumor tissues is significantly correlated with immune infiltration. DCLK1 mRNA expression shows moderate correlation with B cell (**A**) and weak correlation with M1 macrophage (**D**) and no significant correlation with Neutrophil (**B**), CD4+ T cell (**C**), Dendritic cell (**E**) and NK cell (**F**) in tumor tissues of COAD. DCLK1 mRNA expression shows weak correlation with B cell (**G**), CD4+ T cell (**I**) Dendritic cell (**K**) and no significant correlation with Neutrophil (**H**) M1 macrophage (**J**), and NK cell (**L**) in tumor tissues of STAD.

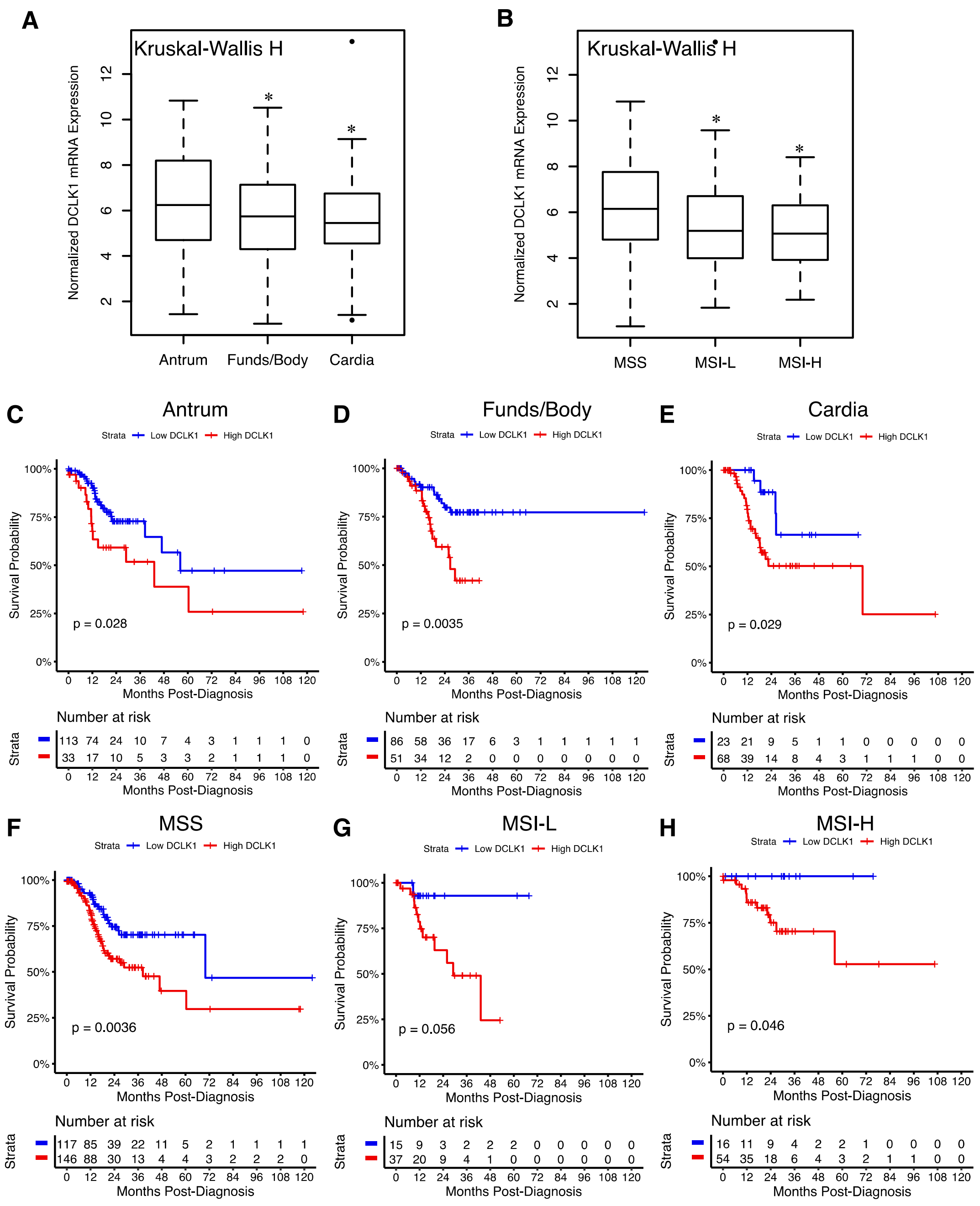

Figure S6. DCLK1 mRNA expression predicts DSS in the patients of every subtype of STAD. (**A**) DCLK1 mRNA expression is higher in patients with tumor originating antrum compared with cardiac and funds or body. No significantly difference shows between patients with tumor originating cardiac and funds or body. * $p < 0.05$ vs. Antrum. (**B**) DCLK1 mRNA expression is higher in microsatellite stable (MSS) subtype compared to microsatellite instable low (MSI-L) or high (MSI-H) subtype. No significantly difference shows between MSI-L and MSI-H subtype. * $p < 0.05$ vs. MSS. Higher DCLK1 mRNA expression predicts worse DSS in patients of with tumor origination antrum (**C**), funds/body (**D**) and cardiac (**E**). Higher DCLK1 mRNA expression predicts worse DSS in MSS (**F**), MSI-L (**G**) and MSI-H (**H**) subtypes of STAD.

Table S1. Clinical patient characteristics for the colon and stomach cancer.

Variable	Colon Cancer	Stomach Cancer	Colon Cancer	Stomach Cancer
Total sample size	283	415		
Age (Mean)	65	65	HR = 1.36 (0.71│2.62) $p = 0.355$	HR = 0.82 (0.55│1.23) $p = 0.339$
>65	125	237		
<65	158	173		
Gender			HR = 1.89 (0.94│3.80) $p = 0.073$	HR = 2.3 (1.32│4.00) $p = 0.481$
Male	127	268		
Female	156	147		
Disease Stage *				
Stage I	45	57		
Stage II (vs. Stage I)	109	123	HR = 1.6 (0.17│14) $p = 0.688$	HR = 2.3 (0.86│6.0) $p = 0.1$
Stage III (vs. Stage I)	80	169	HR = 5.8 (0.73│45) $p = 0.096$	HR = 3.8 (1.51│9.5) $p = 0.005$ *
Stage IV (vs. Stage I)	39	41	HR = 28 (3.37│210) $p = 0.001$ *	HR = 6.3 (2.28│7.3) $p < 0.001$ *
Stage T *				
Stage I + II	50	110		
Stage III (vs. Stage I + II)	193	181	HR = 4.7 (0.63│35) $p = 0.13$	HR = 2.4 (1.4│4.3) $p = 0.003$ *
Stage IV (vs. Stage I + II)	39	115	HR = 34.7 (4.55│264) $p < 0.001$ *	HR = 2.1 (1.1│3.9) $p = 0.024$ *
Nodal Invasion *				
N0	166	123		
N1 (vs. N0)	70	112	HR = 4.0 (1.8-9.2) $p < 0.001$ *	HR = 1.2 (0.66│2.3) $p = 0.509$
N2 (vs. N0)	47	80	HR = 6.4 (.7│15.1) $p < 0.001$ *	HR = 2.0 (1.06│3.6) $p = 0.033$ *
N3 (vs. N0)	0	82	NA	HR = 2.9 (.66│5.2) $p < 0.001$ *
Distant Metastases *				
M0	190	367		
M1 (vs. M0)	39	27	HR = 12.83 (5.73│28.72) $p < 0.001$ *	HR = 2.46 (1.24│4.9) $p = 0.01$ *
MX	50	0		
Tumor Site				
Left	179		HR = 1.14 (0.65│2.68) $p = 0.45$	NA
Right	103			

* $p < 0.05$.

Table S2. Gene list of immune and stromal signatures.

Set	Gene
**Immune Score**	LCP2, LSP1, FYB, PLEK, HCK, IL10RA, LILRB1, NCKAP1L, LAIR1, NCF2, CYBB, PTPRC, IL7R, LAPTM5, CD53, EVI2B, SLA, ITGB2, GIMAP4, MYO1F, HCLS1, MNDA, IL2RG, CD48, AOAH, CCL5, LTB, GMFG, GIMAP6, GZMK, LST1, GPR65, LILRB2, WIPF1, CD37, BIN2, FCER1G, IKZF1, TYROBP, FGL2, FLI1, IRF8, ARHGAP15, SH2B3, TNFRSF1B, DOCK2, CD2, ARHGEF6, CORO1A, LY96, LYZ, ITGAL, TNFAIP3, RNASE6, TGFB1, PSTPIP1, CST7, RGS1,FGR, SELL, MICAL1, TRAF3IP3, ITGA4, MAFB, ARHGDIB, IL4R, RHOH, HLA-DPA1, NKG7, NCF4, LPXN, ITK, SELPLG, HLA-DPB1, CD3D, CD300A, IL2RB, ADCY7, PTGER4, SRGN, CD247, CCR7, MSN, ALOX5AP, PTGER2, RAC2, GBP2, VAV1, CLEC2B, P2RY14, NFKBIA, S100A9, IFI30, MFSD1, RASSF2, TPP1, RHOG, CLEC4A, GZMB, PVRIG, S100A8, CASP1, BCL2A1, HLA-E, KLRB1, GNLY, RAB27A, IL18RAP, TPST2, EMP3, GMIP, LCK, IL32, PTPRCAP, LGALS9, CCDC69, SAMHD1, TAP1, GBP1, CTSS, GZMH, ADAM8, GLRX, PRF1, CD69, HLA-B, HLA-DMA, CD74, KLRK1, PTPRE, HLA-DRA, VNN2, TCIRG1, RABGAP1L, CSTA, ZAP70, HLA-F, HLA-G, CD52, CD302, CD27
**Stromal Score**	DCN, PAPPA, SFRP4, THBS2, LY86, CXCL14, FOXF1, COL10A1, ACTG2, APBB1IP, SH2D1A, SULF1, MSR1, C3AR1, FAP, PTGIS, ITGBL1, BGN, CXCL12, ECM2, FCGR2A, MS4A4A, WISP1, COL1A2, MS4A6A, EDNRA, VCAM1, GPR124, SCUBE2, AIF1, HEPH, LUM, PTGER3, RUNX1T1, CDH5, PIK3R5, RAMP3, LDB2, COX7A1, EDIL3, DDR2, FCGR2B, LPPR4, COL15A1, AOC3, ITIH3, FMO1, PRKG1, PLXDC1, VSIG4, COL6A3, SGCD, COL3A1, F13A1, OLFML1, IGSF6, COMP, HGF, GIMAP5, ABCA6, ITGAM, MAF, ITM2A, CLEC7A, ASPN, LRRC15, ERG, CD86, TRAT1, COL8A2, TCF21, CD93, CD163, GREM1, LMOD1, TLR2, ZEB2, C1QB, KCNJ8, KDR, CD33, RASGRP3, TNFSF4, CCR1, CSF1R, BTK, MFAP5, MXRA5, ISLR, ARHGAP28, ZFPM2, TLR7, ADAM12, OLFML2B, ENPP2, CILP, SIGLEC1, SPON2, PLXNC1,

ADAMTS5, SAMSN1, CH25H, COL14A1, EMCN, RGS4, PCDH12, RARRES2, CD248, PDGFRB, C1QA, COL5A3, IGF1, SP140, TFEC, TNN, ATP8B4, ZNF423, FRZB, SERPING1, ENPEP, CD14, DIO2, FPR1, L18R1, HDC, TXNDC3, PDE2A, RSAD2, ITIH5, FASLG, MMP3, NOX4, WNT2, LRRC32, CXCL9, ODZ4, FBLN2, EGFL6, IL1B, SPON1, CD200

Table S3. Fisher.test of DCLK1 expression in COAD and STAD.

**Variable**	**COAD**			**STAD**		
	N(High)	**N**(Low)	*p*	*N*(High)	*N*(Low)	*p*
**Age (Years)**			0.691			0.058
≥65	43	115		102	135	
<65	37	88		91	82	
**Gender (M v. F)**			1.000			0.413
Female	36	91		65	82	
Male	44	112		130	138	
**Stage (3 + 4 v. 1 + 2)**			0.278			0.127
1 + 2	39	115		75	105	
3 + 4	38	81		104	106	
**Stage T (3 + 4 v. 1 + 2)**			0.169			0.007 *
1 + 2	10	40		39	71	
3 + 4	70	162		150	146	
**Stage N (1+2 v. 0)**			0.081			0.159
0	40	126		51	72	
1 + 2	40	77		135	139	
**Stage M (1 v. 0)**			0.846			
0	54	136		174	193	1.000
1	10	29		13	14	
**Tumor Site (Right v. Left)**			0.217			NA
Left	34	68		NA	NA	
Right	46	133		NA	NA	
**DCLK1(Low v. High)**	80	203		193	217	

* $p < 0.05$.

Table S4. Univariate analysis and multivariate analysis of OS and PFI among COAD according to clinical characteristic and DCLK1 expression.

**Variable**	**OS**									**PFI**								
	Fisher 'Test			Univariate			Multivariate			Fisher 'Test			Univariate			Multivariate		
	H	L	*p*	HR	95% CI	*p*	HR	95% CI	*p*	H	L	*p*	HR	95% CI	*p*	HR	95% CI	*p*
**Age**			0.584	0.68	0.41 | 1.13	0.139	0.43	0.23 | 0.82	0.01 *			0.584	1.30	0.84 | 2.00	0.236	0.96	0.57 | 1.61	0.87
≥65	38	120								38	120							
<65	34	91								34	91							
**Gender**			1.000	0.68	0.91 | 2.42	0.113	1.07	0.60 | 1.92	0.813			1.000	1.48	0.94 | 2.33	0.088	1.16	0.7 | 1.93	0.57
Female	32	95								32	95							
Male	40	116								40	116							
**Stage**			0.483	2.67	1.62 | 4.46	<0.001 *	2.81	0.63 | 12.5	0.175			0.483	2.80	1.77 | 4.14	<0.001	0.9	0.25 | 3.26	0.87
1 + 2	36	118								36	118							
3 + 4	33	86								36	86							
**Stage T**			0.108	2.52	1.01 | 6.26	0.048 *	2.61	0.6 | 11.32	0.2			0.108	3.72	1.51 | 9.2	0.004 *	1.88	0.71 | 4.94	0.2
1 + 2	8	42								8	42							
3 + 4	64	168								64	168							
**Stage N**			0.167	2.49	1.54 | 4.03	<0.001 *	0.6	0.16 | 2.18	0.435			0.167	2.45	1.59 | 3.83	<0.001 *	1.21	0.4 | 3.68	0.74
0	37	129								37	129							
1 + 2	35	82								35	82							
**Stage M**			0.841	4.44	2.5 | 7.9	<0.001 *	3.48	1.67 | 7.26	<0.001 *			0.841	5.90	3.53 | 9.87	<0.001 *	5.71	2.84 | 11.47	<0.001 *
0	49	141								49	141							
1	9	30								9	30							
**Site**			0.476	0.84	0.73 | 1.96	0.481	1.26	0.34 | 1.21	0.43			0.476	0.92	0.59 | 1.44	0.712	1.19	0.72 | 1.99	0.5
Left	34	68								30	79							
Right	46	133								37	120							
**DCLK1**	72	211		0.56	0.34 | 0.94	0.028 *	0.65	0.34 | 1.22	0.18	72	211		0.55	0.35 | 0.88	0.013 *	0.54	0.31 | 0.93	0.024 *

* $p < 0.05$.

Table S5. Univariate analyses and multivariate analyses of OS and PFI among STAD according to clinical characteristic and DCLK1 expression.

**Variable**	**OS**									**PFI**								
	Fisher ‘Test			Univariate			Multivariate			Fisher ‘Test			Univariate			Multivariate		
	H	L	*p*	HR	95% CI	*p*	HR	95% CI	*p*	N(H)	N(L)	*p*	HR	95% CI	*p*	HR	95% CI	*p*
**Age**			1.000	0.65	0.47|0.90	0.01 *	0.58	0.41|0.82	0.002 *			0.058	0.82	0.54|1.23	0.339	0.91	0.63|1.31	0.608
≥65	173	64								102	135							
<65	126	47								91	82							
**Gender**			0.050	1.20	0.86|1.68	0.292	1.22	0.85|1.76	0.28			0.413	1.59	1.00|2.51	0.481	1.82	1.21|2.74	0.004 *
Female	98	49								65	82							
Male	204	64								130	138							
**Stage**			0.056	2.05	1.45|2.9	<0.001*	1.28	0.75|2.16	0.367			0.127	1.67	1.17|2.39	0.005 *	0.93	0.54|1.58	0.784
1 + 2	120	60								75	105							
3 + 4	159	51								104	106							
**Stage T**			<0.001 *	1.85	1.23|2.78	0.226	1.25	0.76|2.04	0.378			0.007 *	1.64	1.05|2.49	0.021 *	1.35	0.82|2.24	0.241
1 + 2	65	45								39	71							
3 + 4	228	68								150	146							
**Stage N**			0.039 *	2.04	1.36|3.06	0.259	1.53	0.86|2.69	0.145			0.156	1.74	1.16|2.61	0.008 *	1.51	0.86|2.63	0.148
0	81	42								51	72							
1 + 2 + 3	208	66								135	139							
**Stage M**			0.658	2.37	1.39|4.05	0.002 *	2.26	1.26|4.06	0.006 *				2.17	1.17|4.03	0.014 *	2.19	1.1|4.38	0.026 *
0	266	101								174	193	1.000						
1	21	6								13	14							
**DCLK1**	302	113	NA	0.50	0.33|0.75	<0.001 *	0.56	0.36|0.86	0.008 *	195	220	NA	0.47	0.33|0.67	<0.001 *	0.5	0.34|0.71	<0.001 *

* $p < 0.05$.

© 2020 by the authors. Licensee MDPI, Basel, Switzerland. This article is an open access article distributed under the terms and conditions of the Creative Commons Attribution (CC BY) license (http://creativecommons.org/licenses/by/4.0/).
